# The Association between Milk Intake and Nutrient Intake Adequacy among Japanese Female Junior High School Students: A Cross-Sectional Study

**DOI:** 10.3390/nu13082838

**Published:** 2021-08-18

**Authors:** Mai Matsumoto, Yoichi Hatamoto, Azusa Sakamoto, Ayumi Masumoto, Chiaki Murayama, Shinji Ikemoto

**Affiliations:** 1 Department of Nutritional Epidemiology and Shokuiku, National Institute of Biomedical Innovation, Health and Nutrition, 1-23-1 Toyama, Shinjuku-ku, Tokyo 162-8636, Japan; 2 Department of Nutrition and Metabolism, National Institutes of Biomedical Innovation, Health and Nutrition, 1-23-1 Toyama, Shinjuku-ku, Tokyo 162-8636, Japan; yhatamoto@nibiohn.go.jp; 3 Department of Registered Dietitian, HANA College of Nutrition, 1-1-12 Negishi, Taitou-ku, Tokyo 110-8662, Japan; azusa_sakamoto_j@yahoo.co.jp; 4 Saitama City, 6-4-4 Tokiwa, Urawa-ku, Saitama-shi, Saitama 330-9588, Japan; ayumimasumoto311@hotmail.com; 5 Department of Human Nutrition, Seitoku University, 550 Iwase, Matsuo-shi, Chiba 271-8555, Japan; chiaki-murayama@hotmail.co.uk

**Keywords:** milk, adolescence, nutritional adequacy, habitual nutrients intake

## Abstract

The consumption of dairy products, including milk, may be important for improving the overall quality of a diet. The aim of this study was to examine the relationship between milk intake and nutritional adequacy among Japanese female junior high school students. The participants of this study were Japanese female junior high school students aged between 12 and 15 years. Dietary habits over the past month were assessed by a brief self-administered diet history questionnaire. The adequacy of each nutrient intake was determined based on the Dietary Reference Intakes for Japanese 2020, with two goals: the estimated average requirement for fourteen nutrients and tentative dietary goals for preventing lifestyle-related diseases for six nutrients. The participants were classified into five groups according to milk intake (Q1 (lowest) to Q5 (highest)) by quintile. The adequacy of vitamin B_2_, calcium, magnesium, and potassium were higher among students with a higher milk consumption than among those with a lower milk consumption. However, the intake of saturated fatty acids was in excess among higher milk consumers. The present study suggests that milk intake was related to an adequate intake of some vitamins and minerals and an inadequate intake of saturated fatty acids among Japanese female junior high school students.

## 1. Introduction

Dietary habits such as diet and dietary behaviours have a major impact on the development of lifestyle-related diseases such as obesity and diabetes [1]. Dietary habits in adolescence also tend to persist into adulthood [2,3,4], which are likely to be difficult to change later in life [5]. In other words, healthy eating patterns including nutritional adequacy established in adolescence may be important to lead to healthy behaviours, prevent chronic diseases including obesity, and decrease morbidity and mortality in adulthood [6]. It is therefore important to identify the factors that influence adolescents’ dietary habits to establish healthy eating habits and to prevent the development of chronic diseases later in life.

The adequate intake of nutrients, including protein, vitamins, and minerals, especially calcium, is important in adolescence because this is a critical period for growth and the build-up of bone mass [7,8]. For this reason, the ingestion of milk and dairy products is recommended because they provide an abundant source of these nutrients, and in particular, calcium [9,10]. Several studies have reported that the consumption of milk and other dairy products may improve the overall nutritional quality including the micronutrient intake among adolescents [11,12]. Additionally, dairy products have been reported to be beneficial for a variety of chronic diseases including metabolic syndrome, type 2 diabetes, hypertension, and cardiovascular disease in adulthood [13,14,15,16]. Moreover, a higher intake of dairy products is associated with a lower prevalence of overweight or obesity among children and adolescents [17]. However, a higher milk consumption has been reported to be related to weight gain and a higher body mass index (BMI) among children and early adolescents [18]. Thus, reports about the association between dairy intake and human health, especially weight gain including being overweight, have been inconsistent. One of the reasons for these inconsistent results may be that milk contains not only a large amount of proteins, vitamins, and minerals, but also a large amount of fats including saturated fatty acids [19]. That is, from the viewpoint of preventing lifestyle-related diseases, it is important to evaluate the relationship between milk intake and the adequacy of nutrient intake with reference to standard values as well as the relationship between milk intake and nutrient intake.

There are few studies about the relationship between milk intake and nutritional adequacy with reference to standard values. For example, a Japanese study reported that the proportion of adults who had a better overall nutritional adequacy, particularly a better calcium intake adequacy, was higher among adults who consumed milk or dairy products than among those who did not consume dairy products [20]. Thus, milk intake may be an important factor for nutritional adequacy and health outcomes. However, there is no study on the association between milk intake and nutritional adequacy among adolescents. More than 80% of the intake of dairy products is from milk in adolescents [12,21]. Additionally, the intake of dairy products decreases during adolescence, and this decrease is primarily attributed to a reduced milk consumption [22]. Although an adequate calcium intake is essential for the development and maintenance of the bone mass peak during adolescence [23], the decrease in dairy products and milk intake may be one of the significant problems. Especially, girls need a large amount of nutrients because they grow considerably during the first half of adolescence [11]. Therefore, it is necessary to assess the association between milk intake and the adequacy of nutrient intake among female junior high school students. The objective of this study was to investigate the association between milk intake and nutritional adequacy among Japanese female junior high school students.

## 2. Materials and Methods

### 2.1. Study Design and Participants

A self-administered diet history questionnaire and a lifestyle questionnaire were distributed by teachers to female junior high school students between 12 and 15 years (*n* = 742) in three junior high schools in the Kanto region, Japan, in June 2016. The questionnaires were completed by the participants themselves, and if necessary, by their parents. The answered questionnaires were checked by the research staff, and any incomplete questionnaires were returned to the students for completion. Five hundred and seventy-five female students completed both questionnaires.

The following respondents were not included: those with missing data (*n* = 17), those who reported milk allergies (*n* = 8), and those with a reported energy intake of less than half of the energy requirement for the lowest physical activity category, according to the Dietary Reference Intake for Japanese (DRIs) [24] or a reported energy intake equal to or more than 1.5 times the energy requirement for the highest physical activity category (<4498 kJ/day or ≥16,945 kJ/day; *n* = 34) [25]. The total number of participants in the final analysis was 516. This sample size was also sufficient because the number of participants required for the logistic regression analysis with the incidence of the inadequacy of nutrient intake (0.15), a significance level (0.05), and statistical power (0.8) was estimated to be a sample of 478 sample in total, by the power analysis using G*Power 3 [26].

The present study was conducted in accordance with the guidelines set out in the Declaration of Helsinki and all the procedures involving human subjects were approved by the Ethics Committee of SEITOKU University, Japan (approval number H27U056). All of the participants and their parents provided written informed consent.

### 2.2. Dietary Assessment

Dietary intake per day over the past month was examined using a brief self-administered diet history questionnaire for Japanese adolescents (BDHQ15y) [26]. The BDHQ15y was developed based on the validated brief self-administered diet history questionnaire for Japanese adults (BDHQ) that asks about dietary history over the past month [27,28]. The BDHQ15y included 67 questions about the intake frequency of foods commonly cooked and consumed in Japan. Daily food, energy, and selected nutrient intakes were calculated using an ad hoc computational algorithm for the BDHQ15y based on the Standard Tables of Food Composition in Japan [29]. The validity of the BDHQ15y was evaluated in the study examining the association between selected food intake and blood biomarker levels [26]. Food groups were categorised as per previous studies [27,30]. Milk was also categorised as either full-fat or low-fat milk.

A self-reported dietary assessment cannot prevent underreporting or over-reporting of food intake [31,32]. Therefore, based on the previous study [33], reported dietary intakes were adjusted to assume that each participant reported the estimated energy requirement (EER) when the physical activity level was at the second level (1.75 times the basal metabolic rate), which is the general physical activity level of adolescence in the Japanese DRIs (2020 Edition) [24]. The following formula was used:

(1) $$\mathrm{dietary}\;\mathrm{intake}\;\left(\mathrm{unit}/\mathrm{day}\right)=\frac{\mathrm{reported}\;\mathrm{dietary}\;\mathrm{intake}\;\left(\mathrm{unit}/\mathrm{day}\right)}{\mathrm{reported}\;\mathrm{energy}\;\mathrm{intake}\;\left(\mathrm{kJ}/\mathrm{day}\right)}\times \mathrm{EER}\;\left(\mathrm{kJ}/\mathrm{day}\right)$$

Intakes of total fat, saturated fatty acids, and carbohydrates were calculated as a percentage of the daily energy intake. In addition, food groups intake values were energy adjusted using the density method (i.e., the amount of the food group per 4184 kJ) to minimise the effect of dietary misreporting.

### 2.3. Determination of the Adequacy of Habitual Nutrient Intake

The adequacy of intake for each nutrient was assessed by a comparison between the nutrient intake levels and each nutrient reference value for the Japanese DRIs (2020 Edition) [24] using the method reported in the previous study [34,35,36]. In the Japanese DRIs [24], the estimated average requirement (EAR) has been set to prevent insufficient nutrient intake and the tentative dietary goal to prevent lifestyle-related diseases (DG) has been set to prevent life style related disease.

For the following 14 nutrients, intakes in excess of the EAR were determined to be adequate using the cut-point method: protein, vitamin A (as retinol activity equivalents), vitamin B_1_, vitamin B_2_, niacin (as niacin equivalents), vitamin B_6_, vitamin B_12_, folate, vitamin C, calcium, magnesium, iron, zinc, and copper. The intake level within the range of the DG values with the following six nutrients was determined to be adequate: total fat, saturated fatty acid, carbohydrate, total dietary fibre, sodium (as salt equivalents), and potassium.

### 2.4. Other Variables

Self-reported body weight and height were asked in BDHQ15y. BMI was calculated as weight (kilograms) divided by the height square (meters). Participants also indicated the weekly frequency of the performance of exercises such as sports club activities over the past month (everyday, 4–6 days/week, 2–3 days/week, one day/week, or never) in the BDHQ15y. Additionally, participants were asked for the following variables by a lifestyle questionnaire: daily breakfast consumption (yes or no), employment status of father (full-time or other), employment status of mother (full-time, part-time or other), frequency of milk consumption at home (everyday, 5–6 days/week, 3–4 days/week, 1–2 days/week, or never), frequency of milk consumption at school lunch (everyday, 3–4 days/week, 1–2 days/week, or never). Participants also reported on their preference for milk by a Likert scale (very like, like, neutral, dislike, very dislike) in the lifestyle questionnaire.

### 2.5. Statistical Analysis

All statistical analyses were conducted using the IBM SPSS statistics software package (version 22.0, SPSS Inc., Chicago, IL, USA). All reported *p*-values were two-tailed with a *p* value of 0.05 considered statistically significant. Participants were classified into five groups by milk intake adjusted using the density method (Q1 (lowest) to Q5 (highest)) by quintile. The differences in the characteristics between the five groups were compared using the chi-square test for categorical variables and the one-way analysis of variance (ANOVA) for continuous variables. Potential confounding factors in the first analysis (model 1) were the employment status of the father (full-time or other) and daily breakfast (yes or no), which was indicated to be substantially different (*p* < 0.05) between the groups categorised according to milk intake. In addition, the confounding factors considered in the second analysis (model 2) were the first model’s factors and the employment status of the mother (full-time, part-time, or other) and the number of days exercising (everyday, 4–6 days/week, 2–3 days/week, one day/week, or never), which was reported to be a factor affecting the nutrient and food consumption among junior high school students in Japan [37]. The nutritional adequacy of each nutrient intake was expressed as a proportion of the participants with intakes above the EAR or within the range of DG in each group. Logistic regression analyses adjusted for the employment status of father and daily breakfast (model 1) and the employment status of father and mother, daily breakfast, and the number of days exercising (model 2) were used to investigate the difference and trends in the prevalence of meeting the DRIs based on the other groups according to the milk intake compared with the lowest group (Q1). Additionally, the trend of daily food group intakes according to the quintile of milk intake was analysed using a regression model adjusted for the confounding variables of the employment status of the father and mother, daily breakfast, and the number of days exercising among 516 Japanese female junior high school students.

## 3. Results

The characteristics of the participants are shown in Table 1. The ranges of milk intake in the five groups were 0–6.34, 6.35–37.28, 37.29–72.92, 72.93–120.31, and 120.32–559.93 g/4184 kJ in Q1 (lowest milk intake) through to Q5 (highest milk intake), respectively. The proportion of people who had breakfast every day was observed in the difference between the five groups. The students who consumed breakfast daily in the highest milk intake group (Q5) accounted for 90.3%, while those of the lowest intake group (Q1) accounted for 78.6%. The proportion of father’s employment status among students was observed in the difference among the five groups (*p* = 0.008); 96% of fathers in the highest milk intake group (Q5) worked full-time. Additionally, the numbers of days drinking milk at home and at school lunch were different among the five groups. Approximately 70% of students in the highest milk intake group had milk intake both at home and at school lunch every day, but more than 80% and approximately half of those in the lowest milk intake group (Q1) had no milk intake at home and at school lunch, respectively. There was a difference in the proportion of the milk preferences among the five groups. The proportion of students liking milk very much was more than 62.1% in the Q5 group, while it was 7.8% in the Q1 group. In addition, the proportion of students who disliked milk very much in the Q1 group was 42.7%. No significant differences were observed in school grade, BMI, habitual exercise status, energy intake, or employment status of the mother among the five groups based on milk intake.

The multivariate-adjusted odds ratios (ORs) for nutrient intake adequacy among female junior high school students are shown in Table 2. The proportion of those having adequate levels of vitamin B_2_ and calcium in the Q3, Q4, and Q5 groups was significantly more than that in the Q1 group (*p* for trend < 0.001). Most of the students in groups Q3–Q5 had adequate levels of vitamin B_2_ (Q3: 97.1%, Q4: 98.1%, Q5: 100%), and the proportion of those with adequate calcium was more than twice that in Q1 (Q1: 35.0%, Q3: 73.1%, Q4: 85.4%, Q5: 91.35%). Additionally, the proportion with an adequate magnesium and potassium intake was higher in groups Q4 and Q5 than in group Q1 (*p* for trend 0.006 and <0.001, respectively). The proportion with an adequate saturated fatty acid intake was lower in groups Q3 and Q5 than in group Q1 (*p* for trend <0.001). The proportion of those with an adequate saturated fatty acid intake in group Q5 (26.2%) was approximately half of that in groups Q1–Q4. Additionally, nearly all the students had adequate intakes of protein, vitamin B_12_, folate, zinc, and copper compared to EAR. On the other hand, there were few students with an adequate intake of vitamin B_1_, iron, and sodium compared to EAR, and dietary fibre compared to DG.

The habitual intake of food groups is shown in Table 3. Grains, breads, noodles, confectioneries, and egg intakes were significantly lower according to milk intake (*p* for trend 0.046, 0.017, 0.007, 0.002, and 0.009, respectively).

## 4. Discussion

The present study investigated the association between milk intake and nutrient intake adequacy among female junior high school students. We found that female junior high school students with a higher milk intake had a higher adequate intake of vitamin B_2_, calcium, magnesium, and potassium, and a lower adequate intake of saturated fatty acid. To the best of our knowledge, the present study is the first study to evaluate the relationship between milk intake and the adequacy of nutrient intakes with reference to the standard values among female junior high school students.

In the current study, the mean milk intake of the female students was approximately 75 g/4184 kJ (150 g/day). Most of the female students in the Q1 group did not consume milk, while the mean amount of milk intake among girls in the Q4 and Q5 groups was approximately 90 g/4184 kJ (200 g/day) and 200 g/4184 kJ (400 g/day), respectively. These results are inconsistent with the mean milk intakes published from other countries including US girls aged 14 years old (400 g/day) [18] and Australian girls aged 12 years old (258 g/day). Furthermore, the milk intake for Japanese adolescents and adults was lower than those of other countries [21,38]. Additionally, the proportion of girls having breakfast everyday was observed in the difference between the five groups; girls with a higher milk consumption were likely to have breakfast every day. This result was similar to the previous study that reported adolescents who frequently had breakfast were milk consumers [39,40].

There was no difference observed in the BMI among the five groups. Additionally, the proportion of obesity in students was not different between the five groups (data not shown). The study of children aged 9–14 years old in the US reported that those who drank more than 3 SV (about 720 mL) of milk a day had a higher BMI than those who did not [18]. Among the participants in the present study, the average milk intake was approximately 400 g/day even in the Q5 group, which had the highest milk intake. This means that the milk intake of Japanese adolescents may have little association with obesity. However, it is necessary to carry out a longitudinal study of the association between milk intake and BMI in Japanese adolescents because this study is a cross-sectional study.

In relation to nutrient intakes, the students with a higher milk intake had adequate intakes of vitamin B_2_, calcium, magnesium, and potassium, especially among the students drinking more than 200 g of milk a day in the Q4 and Q5 groups. Meeting the adequate intake of calcium, magnesium, and potassium has been reported to be difficult without dairy intake [41,42]. These results are similar to the previous study about the positive association between milk intake and the adequate intake of calcium, magnesium, and potassium among Japanese adults [20]. Additionally, it has been reported that vitamin B_2_ is abundant in milk [10]. These studies support the results of the present study which has shown the positive association between milk intake and an adequate nutrient intake. Further study is needed as milk intake may be a predictor of healthy eating behaviour. For saturated fatty acid, excessive intake has been reported to lead to obesity [43], impaired glucose tolerance [43], and cardiovascular disease [44], and it was the only nutrient in which higher milk intake resulted in a higher proportion of inadequate intake in the present study. Milk is high in saturated fatty acids [19], and the previous study for Japanese adults showed the relationship between milk intake and the inadequacy of saturated fatty acids [20]. Thus, milk intake and associated nutrient intakes, such as an adequate intake of minerals and an inadequate intake of saturated fatty acids, are similar in Japanese adolescents and adults. This supports the report of the previous study that dietary habits in adolescence may be difficult to change later in life [19]. That is, milk intake in adolescence may be important in terms of an adequate nutrient intake later in life.

In addition, the milk intake in the present study could be broadly classified into three groups: low milk intake in groups Q1 and Q2, medium milk intake in group Q3, and high milk intake in groups Q4 and Q5. Participants in the Q1 and Q2 groups answered that they did not drink much milk at home or at school lunch, while those in the Q3 group drank as much as the students in the Q4 and Q5 groups at school lunch but they tended to consume milk less frequently at home. Moreover, in the Q3 group, the intake of confectioneries was higher than those in the Q5 group. The timing of milk intake for children and adolescents in the US is reported to be at breakfast or in the afternoon [40]. In other words, milk may be consumed as a snack in addition to being consumed at breakfast for adolescents. Additionally, protein intake from dairy products has been reported to be satisfying and may reduce appetites among Europeans and Australians [45]. Thus, the students in the Q4 and Q5 groups might drink milk as a snack, which might result in a reduction in confectionery intake, but it may be possible that the intake of saturated fatty acids increased because the frequency of drinking milk at home was reduced and the intake of confectionery increased in the Q3 group. This may be related to the higher proportion of students with an inadequate intake of saturated fatty acids in the Q3 group than in the Q5 group. In the future, it may be necessary to consider the dietary patterns at home to assess the relationship between milk intake and saturated fatty acid intake. Additionally, milk contains a large amount of vitamins and minerals as well as saturated fatty acid, while low-fat milk is recommended as a healthy food because it contains more vitamins and minerals but less saturated fatty acid [46]. Among the participants in this study, more than half of those in each group did not drink low-fat milk (median low-fat milk intake in all five groups: 0 g/day, data not shown). Further research is needed, for example on the association between fat concentrations in milk and individuals’ satisfaction, but it may also be necessary to consider preventing an excessive intake of saturated fatty acid by means of recommending low-fat milk intake.

There was little to no nutrient inadequacy for protein, vitamin B_12_, folate, zinc, or copper among most of the participants. It has been reported that these nutrients are scarcely deficient in Japanese adolescents [33,47] and women [35,48], and thus, these reports are consistent with those of the present study. Meanwhile, vitamin B_1_, iron, and dietary fibre were deficient among the students. These results are consistent with previous studies which have reported deficiencies of these nutrients among Japanese adolescents and women [47,48]. In other words, protein, vitamin B_12_, and copper may be nutrients which are adequately consumed by most Japanese people, and vitamin B_1_, iron, dietary fibre, and sodium may be nutrients to take note of regarding intake for many Japanese people.

Among the participants in this study, the intake of grains including bread and noodles decreased as milk intake increased. This result is different from a previous report which showed a positive association between bread and milk intakes in the dietary pattern of Japanese adults [49]. However, in Japan, it is reported that 88.1% of junior high school students drink milk every day at school lunch, regardless of whether the staple food is rice or bread [50]. This may partly explain the results of the present study. However, the results of the present study were those of habitual food group consumption, and the relationship between staple food and milk intake at each mealtime has not been examined. Since the timing of milk intake is not assessed in the present study, the relationship with grains, which is a staple food, cannot be assessed. It may be necessary to consider the association between milk intake and grains as a staple food after considering the timing of milk intake among Japanese junior high school students.

There are several limitations to this study. First, the participants in the present study were not sampled randomly, and the survey area was limited to a single region in Japan. Therefore, the participants were probably not representative of Japanese adolescents. Second, the ability of BDHQ15y to estimate dietary intake has only been validated with a limited number of foods and nutrients [26]. Third, the intake of dietary supplements was not included in the analysis because reliable composition tables of dietary supplements were lacking in Japan. Fourth, the household income, education, and nutritional habits of the participants’ parents were not examined. These factors have been reported to affect the diets of children [51,52]. The employment status of both parents was investigated in this study, but this information does not necessarily correspond to household income. Fifth, the amount of physical activity for each individual was not investigated. Therefore, we used the second physical activity level for the calculation of EER [24], and we cannot deny the possibility that this may have affected the results. However, this value has been used in previous studies for the same age group as the present study to adjust nutrient intakes with EER when physical activity was unknown [33]. Finally, we did not examine the experience of participant’s first menstruation. The EAR for iron changes depending on menstruation in females. A previous study reported that 585 out of 4769 female junior high school students (12.3%) had not experienced their first menstruation, and the majority of these were 12 years old [53]. Because female junior high school students are likely to have their first menstruation soon, if not already, they require a higher iron intake. Therefore, the EAR for iron for females who are menstruating was considered as a more appropriate reference value to evaluate nutritional adequacy in the present study.

## 5. Conclusions

The present cross-sectional study showed that milk intake was related to the adequate intake of vitamin B_2_ and some minerals such as calcium, magnesium, and potassium, and the inadequate intake of saturated fatty acid among Japanese female junior high school students. Milk may be an important source of nutrition for Japanese female adolescents; however, it may be beneficial to promote the intake of low-fat milk to prevent the intake of excess saturated fatty acids while consuming a large quantity of milk.

## Figures and Tables

**Table 1:** Characteristics of 516 Japanese female junior high school students classified into five groups according to milk intake.

	All (*n* = 516)		Q1 (Lowest) (*n* = 103)		Q2 (*n* = 103)		Q3 (*n* = 104)		Q4 (*n* = 103)		Q5 (Highest) (*n* = 103)		*p* *
Milk intake (g/4184 kJ), Mean (SD)	75.0	(80.0)	1.1	(2.1)	19.8	(9.3)	56.2	(10.0)	91.0	(12.9)	204.8	(75.9)	<0.001
School grade, *n* (%)													0.384
1	176	(34.1)	35	(34.0)	34	(33.0)	38	(36.5)	30	(29.1)	39	(37.9)	
2	187	(36.2)	29	(28.2)	40	(38.8)	42	(40.4)	40	(38.8)	36	(35.0)	
3	153	(29.7)	39	(37.9)	29	(28.2)	24	(23.1)	33	(32.0)	28	(27.2)	
Body height (cm), Mean (SD)	154.5	(7.8)	153.8	(7.1)	154.4	(5.8)	155.9	(5.5)	154.9	(6.8)	153.3	(12.0)	0.150
Body weight (kg), Mean (SD)	45.7	(7.5)	44.8	(7.3)	44.9	(6.9)	47.2	(8.6)	46.2	(7.6)	45.3	(7.0)	0.116
Body mass index (kg/m^2^), Mean (SD)	19.3	(6.0)	18.9	(2.4)	18.8	(2.2)	19.4	(2.9)	19.2	(2.5)	20.2	(12.4)	0.137
Number of days exercising, *n* (%)													0.091
Everyday	231	(44.8)	43	(41.7)	34	(33.0)	50	(48.1)	46	(44.7)	58	(56.3)	
4–6 days/week	78	(15.1)	13	(12.6)	23	(22.3)	15	(14.4)	12	(11.7)	15	(14.6)	
2–3 days/week	61	(11.8)	13	(12.6)	18	(17.5)	9	(8.7)	15	(14.6)	6	(5.8)	
1 day/week	49	(9.5)	9	(8.7)	13	(12.6)	10	(9.6)	10	(9.7)	7	(6.8)	
Never	97	(18.8)	25	(24.3)	15	(14.6)	20	(19.2)	20	(19.4)	17	(16.5)	
Daily breakfast, *n* (%)													0.015
Yes	427	(82.8)	81	(78.6)	76	(73.8)	90	(86.5)	87	(84.5)	93	(90.3)	
No	89	(17.2)	22	(21.4)	27	(26.2)	14	(13.5)	16	(15.5)	10	(9.7)	
Energy intake (kJ/day), Mean (SD)	8799	(2289)	8146	(1908)	8439	(2310)	10,075	(2084)	8272	(2247)	9063	(2347)	0.518
Employment status of father, *n* (%)													0.008
Full-time	445	(86.2)	90	(87.4)	87	(84.5)	82	(78.8)	87	(84.5)	99	(96.1)	
Other	71	(13.8)	13	(12.6)	16	(15.5)	22	(21.2)	16	(15.5)	4	(3.9)	
Employment status of mother, *n* (%)													0.209
Full-time	109	(21.1)	21	(20.4)	29	(28.2)	20	(19.2)	20	(19.4)	19	(18.4)	
Part-time	204	(39.5)	47	(45.6)	38	(36.9)	35	(33.7)	47	(45.6)	37	(35.9)	
Other	203	(39.3)	35	(34.0)	36	(35.0)	49	(47.1)	36	(35.0)	47	(45.6)	
Frequency of milk consumption at home, *n* (%)													<0.001
Everyday	120	(23.3)	1	(1.0)	4	(3.9)	17	(16.3)	29	(28.2)	69	(67.0)	
5–6 days/week	38	(7.4)	0	(0.0)	0	(0.0)	11	(10.6)	15	(14.6)	12	(11.7)	
3–4 days/week	63	(12.2)	3	(2.9)	17	(16.5)	23	(22.1)	11	(10.7)	9	(8.7)	
1–2 days/week	108	(20.9)	10	(9.7)	53	(51.5)	24	(23.1)	16	(15.5)	5	(4.9)	
Never	187	(36.2)	89	(86.4)	29	(28.2)	29	(27.9)	32	(31.1)	8	(7.8)	
Frequency of milk consumption at school at lunch, *n* (%)													<0.001
Everyday	272	(52.7)	24	(23.3)	35	(34.0)	69	(66.3)	71	(68.9)	73	(70.9)	
3–4 days/week	21	(4.1)	2	(1.9)	4	(3.9)	9	(8.7)	4	(3.9)	2	(1.9)	
1–2 days/week	101	(19.6)	28	(27.2)	33	(32.0)	13	(12.5)	15	(14.6)	12	(11.7)	
Never	122	(23.6)	49	(47.6)	31	(30.1)	13	(12.5)	13	(12.6)	16	(15.5)	
Preference for milk, *n* (%)													<0.001
Very like	145	(28.1)	8	(7.8)	23	(22.3)	24	(23.1)	26	(25.2)	64	(62.1)	
Like	132	(25.6)	12	(11.7)	34	(33.0)	33	(31.7)	30	(29.1)	23	(22.3)	
Neutral	113	(21.9)	24	(23.3)	25	(24.3)	29	(27.9)	27	(26.2)	8	(7.8)	
Dislike	57	(11.0)	15	(14.6)	13	(12.6)	13	(12.5)	11	(10.7)	5	(4.9)	
Very dislike	69	(13.4)	44	(42.7)	8	(7.8)	5	(4.8)	9	(8.7)	3	(2.9)	

SD, standard deviation. * The *p* values are shown for chi-square test for categorical variables and for one-way analysis of variance for continuous variables to analyse differences among quintile of milk intake of 516 female junior high school students.

**Table 2:** Daily nutrient intakes and prevalence of meeting EAR and DG according to quintile of milk intake among 516 female junior high school students ^†^.

	Reference Value ^‡^	All [Mean (SD)]		Adequacy ^§^ (%)	Q1 (Lowest) [Mean (SD)]		Adequacy ^§^ (%)	Q2 [Mean (SD)]		Adequacy ^§^ (%)	Q3 [Mean (SD)]		Adequacy ^§^ (%)	Q4 [Mean (SD)]		Adequacy ^§^ (%)	Q5 (Highest) [Mean (SD)]		Adequacy ^§^ (%)	*p* ^§§^
*n*		516			103			103			104			103			103			
Nutrient with EAR																				
Protein (g)	≥45	80	(14)	100	77	(13)	100	81	(17)	100	78	(12)	100	81	(11)	100	86	(13)	100	
Model 1: OR (95% CI) ^††^							1.00 (Reference)			-			-			-			-	-
Model 2: OR (95% CI) ^‡‡^							1.00 (Reference)			-			-			-			-	-
Vitamin A (µgRAE) ^ǁ^	≥500	804	(395)	82.2	795	(467)	78.6	811	(498)	74.8	768	(276)	81.7	790	(318)	89.3	858	(375)	87.4	
Model1: OR (95% CI) ^††^							1.00 (Reference)			0.82 (0.43–1.57)			1.17 (0.59–2.33)			2.01 (0.93–4.33)			1.80 (0.84–3.82)	0.096
Model2: OR (95% CI) ^‡‡^							1.00 (Reference)			0.71 (0.36–1.38)			1.07 (0.53–2.16)			1.91 (0.88–4.15)			1.75 (0.81–3.77)	0.064
Vitamin B_1_ (mg)	≥1.1	0.95	(0.17)	19.8	0.92	(0.17)	17.5	0.93	(0.19)	17.5	0.93	(0.15)	15.4	0.96	(0.15)	18.4	1.0	(0.16)	25.2	
Model 1: OR (95% CI) ^††^							1.00 (Reference)			1.04 (0.50–2.13)			0.83 (0.40–1.74)			1.04 (0.51–2.12)			1.48 (0.75–2.93)	0.573
Model 2: OR (95% CI) ^‡‡^							1.00 (Reference)			0.98 (0.47–2.05)			0.76 (0.36–1.62)			1.00 (0.48–2.06)			1.38 (0.69–2.77)	0.588
Vitamin B_2_ (mg)	≥1.2	1.7	(0.42)	91.9	1.5	(0.40)	82.5	1.6	(0.42)	81.6	1.7	(0.28)	97.1	1.8	(0.31)	98.1	2.1	(0.40)	100	
Model 1: OR (95% CI) ^††^							1.00 (Reference)			0.95 (0.48–2.01)			7.20 (2.03–25.6)			10.6 (2.38–47.2)			–	0.001
Model 2: OR (95% CI) ^‡‡^							1.00 (Reference)			0.93 (0.44–1.97)			7.83 (2.16–28.4)			10.6 (2.34–47.8)			–	0.001
Niacin (mgNE) ^¶^	≥12	17	(4.2)	89.2	17	(3.7)	90.3	18	(5.8)	88.3	16	(3.5)	88.5	17	(3.5)	89.3	16	(4.0)	84.5	
Model 1: OR (95%CI)^††^							1.00 (Reference)			0.84 (0.34–2.05)			0.77 (0.31–1.88)			0.86 (0.35–2.13)			0.55 (0.23–1.29)	0.685
Model 2: OR (95% CI) ^‡‡^							1.00 (Reference)			0.75 (0.30–1.87)			0.80 (0.32–1.98)			0.84 (0.33–2.10)			0.58 (0.25–1.39)	0.805
Vitamin B_6_ (mg)	≥1.0	1.3	(0.32)	79.8	1.3	(0.32)	74.8	1.4	(0.40)	78.6	1.3	(0.26)	79.8	1.4	(0.28)	86.4	1.4	(0.10)	79.6	
Model 1: OR (95% CI) ^††^							1.00 (Reference)			1.22 (0.52–2.88)			1.18 (0.50–2.78)			1.18 (0.50–2.78)			1.31 (0.54–3.17)	0.981
Model 2: OR (95% CI) ^‡‡^							1.00 (Reference)			1.17 (0.49–2.80)			1.22 (0.51–2.92)			1.16 (0.51–2.92)			1.42 (0.58–3.47)	0.964
Vitamin B_12_ (mg)	≥2.0	8.4	(3.9)	99.8	7.9	(4.1)	100	8.7	(4.5)	100	8.1	(3.4)	99.0	(8.8)	(3.6)	100	8.8	(3.8)	100	
Model 1: OR (95% CI) ^††^							1.00 (Reference)			-			-			-			-	-
Model 2: OR (95% CI) ^‡‡^							1.00 (Reference)			-			-			-			-	-
Folate (µg)	≥200	395	(136)	96.7	407	(155)	94.2	398	(166)	96.2	379	(106)	97.1	398	(126)	99.0	393	(121)	98.1	
Model 1: OR (95% CI) ^††^							1.00 (Reference)			0.90 (0.31–2.61)			2.46 (0.61–9.93)			3.61 (0.73–17.9)			2.10 (0.52–8.50)	0.271
Model 2: OR (95% CI) ^‡‡^							1.00 (Reference)			0.92 (0.31–2.73)			2.25 (0.55–9.21)			3.48 (0.69–17.5)			1.98 (0.48–8.18)	0.358
Vitamin C (mg)	≥85	135	(56)	85.9	139	(59)	87.4	132	(60)	79.6	133	(49)	86.5	137	(57)	88.3	133	(55)	87.4	
Model1: OR (95% CI) ^††^							1.00 (Reference)			0.57 (0.27–1.19)			0.66 (0.31–1.42)			0.68 (0.31–1.45)			0.69 (0.31–1.46)	0.673
Model 2: OR (95% CI) ^‡‡^							1.00 (Reference)			0.51 (0.24–1.09)			0.60 (0.28–1.31)			0.61 (0.28–1.33)			0.64 (0.29–1.41)	0.533
Calcium (mg)	≥700	856	(286)	67.1	673	(238)	35.0	725	(241)	43.7	828	(194)	73.1	897	(193)	85.4	1159	(276)	98.1	
Model 1: OR (95% CI) ^††^							1.00 (Reference)			1.51 (0.86–2.67)			4.99 (2.73–9.10)			11.0 (5.52–21.8)			88.9 (20.7–382.7)	<0.001
Model 2: OR (95% CI) ^‡‡^							1.00 (Reference)			1.38 (0.77–2.49)			4.92 (2.66–9.09)			10.9 (5.46–21.9)			91.7 (21.2–397.0)	<0.001
Magnesium (mg)	≥240	286	(56)	80.0	273	(57)	69.9	285	(68)	74.8	278	(44)	80.8	291	(50)	83.5	305	(55)	91.3	
Model 1: OR (95% CI) ^††^							1.00 (Reference)			1.29 (0.70–2.38)			1.89 (0.98–3.62)			2.22 (1.13–4.35)			4.39 (1.96–9.85)	0.003
Model 2: OR (95% CI) ^‡‡^							1.00 (Reference)			1.28 (0.68–2.41)			1.91 (0.98–3.73)			2.17 (1.09–4.29)			4.74 (2.08–10.8)	0.003
Iron (mg)	≥10.0	8.8	(2.1)	25.0	9.2	(2.3)	32.0	9.1	(2.5)	26.2	8.6	(1.7)	23.1	8.8	(1.9)	25.2	8.3	(1.8)	18.4	
Model 1: OR (95% CI) ^††^							1.00 (Reference)			0.78 (0.42–1.43)			0.61 (0.33–1.13)			0.69 (0.37–1.27)			0.44 (0.23–0.85)	0.162
Model 2: OR (95%CI) ^‡‡^							1.00 (Reference)			0.69 (0.37–1.30)			0.56 (0.30–1.07)			0.63 (0.34–1.17)			0.44 (0.22–0.85)	0.159
Zinc (mg)	≥7.0	9.9	(1.4)	98.4	9.5	(1.4)	97.1	9.9	(1.6)	98.1	9.5	(1.2)	99.0	9.9	(1.2)	98.1	10.5	(1.2)	100.0	
Model 1: OR (95% CI) ^††^							1.00 (Reference)			1.65 (0.27–10.3)			2.78 (0.28–27.9)			1.36 (0.22–8.49)			-	0.933
Model 2: OR (95% CI) ^‡‡^							1.00 (Reference)			2.40 (0.35–16.7)			5.50 (0.43–70.2)			2.04 (0.29–14.2)			-	0.747
Copper (mg)	≥0.6	1.3	(0.21)	100	1.3	(0.23)	100	1.3	(0.24)	100	1.2	(0.17)	100	1.3	(0.19)	100	1.2	(0.21)	100	
Model 1: OR (95% CI) ^††^							1.00 (Reference)			-			-			-			-	-
Model 2: OR (95% CI) ^‡‡^							1.00 (Reference)			-			-			-			-	-
Nutrient with DG																				
Fat (% energy)	20–30	30.2	(5.4)	44.2	30.0	(5.9)	45.6	28.9	(5.9)	48.5	30.8	(5.1)	43.4	30.5	(5.0)	42.7	30.8	(5.0)	40.8	
Model 1: OR (95% CI) ^††^							1.00 (Reference)			1.12 (0.65–1.93)			0.94 (0.54–1.62)			0.90 (0.52–1.57)			0.83 (0.48–1.45)	0.878
Model 2: OR (95% CI) ^‡‡^							1.00 (Reference)			1.17 (0.67–2.05)			0.99 (0.56–1.73)			0.95 (0.55–1.66)			0.85 (0.48–1.50)	0.871
Saturated fatty acid (% energy)	<10.0	10.2	(2.5)	48.3	9.7	(2.7)	58.3	9.1	(2.3)	67.0	10.6	(2.2)	41.3	10.4	(2.3)	48.5	11.4	(2.2)	26.2	
Model 1: OR (95% CI) ^††^							1.00 (Reference)			1.43 (0.81–2.52)			0.52 (0.30–0.91)			0.69 (0.40–1.20)			0.27 (0.15–0.48)	<0.001
Model 2: OR (95% CI) ^‡‡^							1.00 (Reference)			1.49 (0.83–2.68)			0.54 (0.31–0.95)			0.71 (0.41–1.25)			0.27 (0.15–0.49)	<0.001
Carbohydrate (% energy)	50–65	53.7	(6.6)	67.4	54.7	(7.1)	68.9	54.6	(7.4)	68.9	53.5	(6.3)	64.4	53.3	(5.8)	70.9	52.3	(6.3)	64.1	
Model 1: OR (95% CI) ^††^							1.00			1.02 (0.56–1.84)			0.84 (0.47–1.50)			1.10 (0.61–2.01)			0.77 (0.43–1.38)	0.738
Model 2: OR (95% CI) ^‡‡^							(Reference)			1.06 (0.58–1.93)			0.88 (0.49–1.60)			1.13 (0.61–2.07)			0.81 (0.44–1.46)	0.817
Total dietary fiber (g)	≥17	13.1	(3.8)	19.8	13.8	(4.1)	27.2	13.4	(4.5)	19.4	12.7	(3.2)	11.5	13.1	(3.5)	20.4	12.4	(3.5)	15.5	
Model 1: OR (95% CI) ^††^							1.00 (Reference)			0.85 (0.41–1.78)			0.36 (0.15–0.88)			0.74 (0.35–1.55)			0.47 (0.21–1.06)	0.128
Mode l2: OR (95% CI) ^‡‡^							1.00 (Reference)			0.73 (0.34–1.59)			0.32 (0.13–0.79)			0.71 (0.33–1.53)			0.44 (0.19–1.01)	0.101
Sodium (salt-equivalent) (g)	<6.5	12.2	(2.5)	1.0	12.7	(2.4)	0	12.8	(2.9)	1.9	11.6	(2.5)	1.9	12.5	(2.2)	0	11.6	(2.2)	1.0	
Model 1: OR (95% CI) ^††^							1.00 (Reference)			-			-			-			-	0.968
Model 2: OR (95% CI) ^‡‡^							1.00 (Reference)			-			-			-			-	0.946
Potassium (mg)	≥2400	2801	(698)	69.0	2639	(684)	57.3	2669	(816)	55.3	2707	(554)	67.3	2836	(637)	74.8	3156	(656)	92.3	
Model 1: OR (95% CI) ^††^							1.00 (Reference)			0.95 (0.55–1.66)			1.59 (0.89–2.82)			2.23 (1.23–4.05)			6.42 (3.00–13.8)	<0.001
Model 2: OR (95% CI) ^‡‡^							1.00 (Reference)			0.91 (0.51–1.60)			1.58 (0.88–2.83)			2.17 (1.18–3.97)			6.79 (3.13–14.8)	<0.001

EAR, estimated average requirement; DG, tentative dietary goal for preventing lifestyle-related disease; SD, standard deviation; CI, confidence interval; DRI, Dietary Reference Intakes; OR, odd ratio. ^†^ Adjustment of the reporting error was performed according to the following: Nutrient intake = reported nutrient intake/reported energy intake × estimated energy requirement. ^‡^ DRIs for 12- to 14-year-old Japanese girls. The estimated energy requirement for physical activity level Ⅱ is 10,042 kJ/day. ^§^ Percentage of participants whose nutrient intake met DG or EAR of DRIs. Each nutrient intake was compared with each DRI value, using the cut-point method. ^ǁ^ Sum of retinol, β-carotene/12, α-carotene/24, and cryptoxanthin/24. ^¶^ Sum of niacin and protein/6000. ^††^ Multivariate adjusted ORs on nutrient intake adequacy of the Q2 to Q5 groups with reference to the Q1 group were calculated by adjusting for the employment status of father (full-time or others) and daily breakfast (yes or no). ^‡‡^ Multivariate adjusted ORs on the nutrient intake adequacy of the Q2 to Q5 groups with reference to the Q1 group were calculated by adjusting for the employment status of father (full-time or others) and mother (full-time, part-time or others), daily breakfast (yes or no) and the number of days exercising (everyday, 4–6 days/week, 2–3 days/week, one day/week, or never). ^§§^ The *p* values are shown for the trend test of the logistic regression model adjusted for confounding variables (model 1: employment status of father and daily breakfast, model 2: e status of father and mother, daily breakfast, the number of days exercising) to assess the trend for nutrient intake adequacy according to the milk intake of 516 junior high school students.

**Table 3:** Daily food group intakes according to quintile of milk intake of 516 female junior high school students (g/4184 kJ) ^†^.

	Q1 (Lowest) (*n* = 103)		Q (*n* = 103)		Q3 (*n* = 104)		Q4 (*n* = 103)		Q5 (Highest) (*n* = 103)		*p* ^‡^
Grains	212.1	(66.0)	230.5	(73.6)	196.2	(65.1)	209.4	(53.9)	195.7	(54.0)	0.046
Rice	156.1	(71.9)	180.5	(75.6)	145.7	(68.9)	160.9	(59.0)	153.2	(59.1)	0.583
Bread	24.6	(14.8)	18.5	(12.9)	22.5	(12.5)	21.9	(13.7)	19.3	(12.2)	0.017
Noodles	31.4	(23.0)	31.6	(26.8)	28.0	(22.4)	26.6	(18.2)	23.2	(15.4)	0.007
Pulses	29.0	(20.1)	34.3	(23.3)	29.3	(15.5)	30.9	(19.2)	29.0	(17.7)	0.461
Potatoes	16.5	(9.6)	17.7	(10.2)	16.4	(10.5)	13.9	(9.8)	14.5	(12.3)	0.226
Sugar	2.9	(3.3)	2.4	(1.9)	2.7	(2.4)	2.4	(2.0)	2.2	(1.8)	0.081
Confectioneries	54.1	(33.2)	40.5	(26.3)	53.2	(27.7)	38.6	(24.0)	33.0	(23.4)	0.002
Fat and oil	8.2	(3.7)	8.0	(3.0)	7.5	(3.1)	8.1	(3.0)	7.0	(3.4)	0.162
Fat	0.7	(1.1)	0.6	(0.9)	1.0	(0.7)	0.6	(0.9)	0.5	(0.8)	0.218
Oil	7.5	(3.8)	7.4	(2.7)	6.9	(2.8)	7.5	(2.7)	6.5	(3.1)	0.268
Fruits	31.8	(32.8)	32.7	(30.2)	34.7	(28.8)	37.0	(35.6)	35.6	(32.8)	0.782
Total vegetables	136.7	(84.2)	131.5	(90.1)	115.9	(57.1)	123.7	(64.6)	123.0	(66.4)	0.722
Green and yellow vegetables	52.0	(37.6)	47.4	(35.5)	43.4	(25.2)	46.2	(30.1)	46.0	(27.5)	0.805
Other vegetables	68.5	(45.8)	68.7	(47.6)	58.6	(31.4)	62.0	(35.8)	60.9	(36.5)	0.439
Pickled vegetables	5.4	(7.3)	5.5	(6.2)	4.6	(5.3)	5.1	(5.4)	4.5	(6.3)	0.799
Mushrooms	4.8	(5.6)	4.5	(5.2)	4.3	(3.9)	4.7	(3.8)	5.0	(5.4)	0.318
Seaweeds	6.0	(5.6)	5.3	(5.9)	5.0	(4.2)	5.6	(4.2)	6.6	(6.5)	0.090
Beverages	348.1	(184.9)	306.1	(186.8)	322.4	(165.1)	333.9	(195.4)	298.3	(159.4)	0.584
Fruit and vegetable juice	35.0	(60.8)	23.2	(39.8)	35.8	(50.6)	28.4	(41.5)	26.8	(43.8)	0.301
Green tea	214.9	(138.3)	182.9	(138.4)	190.8	(105.2)	207.7	(132.3)	201.0	(121.8)	0.452
Black tea	34.7	(83.8)	53.4	(92.8)	40.8	(87.8)	41.4	(79.4)	28.2	(69.8)	0.239
Soft drinks	63.4	(87.1)	46.5	(63.6)	55.0	(84.1)	56.4	(91.2)	42.2	(56.2)	0.545
Fish and shellfish	30.3	(17.4)	34.6	(21.2)	29.6	(15.8)	31.3	(15.6)	28.9	(17.0)	0.085
Meat	40.3	(17.5)	41.5	(21.7)	37.1	(17.2)	37.2	(13.5)	36.0	(15.7)	0.961
Eggs	20.0	(13.1)	19.2	(10.3)	17.8	(9.7)	18.1	(10.1)	16.2	(8.5)	0.009
Dairy products	39.8	(45.3)	72.2	(55.0)	114.1	(44.2)	144.7	(45.1)	263.6	(90.7)	<0.001

SD, standard deviation, the values are presented as mean (SD). ^†^ Adjustment of reporting error was performed according to the following: Food group intake = reported food group intake/reported energy intake × 4184 (kJ). ^‡^ The *p* values are shown for the trend test of the regression model adjusted for the confounding variables of employment status of father (full-time or others) and mother (full-time, part-time or others), daily breakfast (yes or no), the number of days exercising (everyday, 4–6 days/week, 2–3 days/week, 1 day/week, or never) to analyze the trend for food groups intake according to quintile of milk intake in 516 junior high school students.
